# Gut Microbiota and NAFLD: Pathogenetic Mechanisms, Microbiota Signatures, and Therapeutic Interventions

**DOI:** 10.3390/microorganisms9050957

**Published:** 2021-04-29

**Authors:** Tomas Hrncir, Lucia Hrncirova, Miloslav Kverka, Robert Hromadka, Vladimira Machova, Eva Trckova, Klara Kostovcikova, Pavlina Kralickova, Jan Krejsek, Helena Tlaskalova-Hogenova

**Affiliations:** 1 Czech Academy of Sciences, Institute of Microbiology, 142 20 Prague, Czech Republic; lucia.hrncirova@gmail.com (L.H.); kverka@biomed.cas.cz (M.K.); vladimira.machova@biomed.cas.cz (V.M.); eva.trckova@centrum.cz (E.T.); klimesov@biomed.cas.cz (K.K.); tlaskalo@biomed.cas.cz (H.T.-H.); 2 The Faculty of Medicine in Hradec Kralove, Charles University in Prague, 500 03 Hradec Kralove, Czech Republic; pavlina.kralickova@fnhk.cz (P.K.); jan.krejsek@fnhk.cz (J.K.); 3 NEXARS (C2P), The Campus Science Park, 625 00 Brno, Czech Republic; hromadka@nexars.com

**Keywords:** liver steatosis, cirrhosis, hepatocellular carcinoma, intestinal permeability, gut microbiota dysbiosis, loss of diversity, faecal microbiota transplantation

## Abstract

Non-alcoholic fatty liver disease (NAFLD) is the most common chronic liver disease. Its worldwide prevalence is rapidly increasing and is currently estimated at 24%. NAFLD is highly associated with many features of the metabolic syndrome, including obesity, insulin resistance, hyperlipidaemia, and hypertension. The pathogenesis of NAFLD is complex and not fully understood, but there is increasing evidence that the gut microbiota is strongly implicated in the development of NAFLD. In this review, we discuss the major factors that induce dysbiosis of the gut microbiota and disrupt intestinal permeability, as well as possible mechanisms leading to the development of NAFLD. We also discuss the most consistent NAFLD-associated gut microbiota signatures and immunological mechanisms involved in maintaining the gut barrier and liver tolerance to gut-derived factors. Gut-derived factors, including microbial, dietary, and host-derived factors involved in NAFLD pathogenesis, are discussed in detail. Finally, we review currently available diagnostic and prognostic methods, summarise latest knowledge on promising microbiota-based biomarkers, and discuss therapeutic strategies to manipulate the microbiota, including faecal microbiota transplantation, probiotics and prebiotics, deletions of individual strains with bacteriophages, and blocking the production of harmful metabolites.

## 1. Introduction

Non-alcoholic fatty liver disease (NAFLD) is characterised by an excessive intrahepatic fat accumulation, i.e., steatosis, without significant alcohol consumption. Liver steatosis is defined as fat accumulation, in >5% of hepatocytes [1]. NAFLD may be present in several forms ranging from simple steatosis to non-alcoholic steatohepatitis (NASH), which is a progressive form, characterised by steatosis, hepatocytes swelling, and inflammation. Unlike simple steatosis, NASH is not reversible and can eventually progress into fibrosis, cirrhosis, or even hepatocellular carcinoma (HCC).

The first stage of alcoholic liver disease (ALD) is also characterised by hepatic steatosis. However, unlike NAFLD, the primary trigger of ALD, i.e., excessive alcohol consumption, is known and the disease is preventable. Ethanol probably does not play a prominent role in NAFLD pathogenesis but is discussed as one of the possible contributing factors. A detailed discussion of the role of the gut microbiota in ALD pathogenesis is beyond the scope of this review and has been discussed elsewhere [2].

NAFLD is closely associated with many features of metabolic syndrome, including obesity, insulin resistance, hyperlipidaemia, and hypertension [3,4] and increases the risk of cardiovascular disease (CVD) and type 2 diabetes mellitus (T2DM) [5]. Therefore, not surprisingly, the leading cause of death in NAFLD patients is not liver failure, but cardiovascular disease [6].

NAFLD is the most common chronic liver condition in the USA and Europe. Its global prevalence is rapidly increasing and is currently estimated at 24%. The highest rates are reported from the Middle East (32%) and South America (31%) and the lowest from Africa (14%) [7]. The estimated 10-year economic burden of NAFLD alone could increase to an estimated USD 1.005 trillion in the USA and EUR 334 billion in Europe [8].

NAFLD pathogenesis is complex and not fully understood. The current understanding is that NAFLD is caused by a complex interplay of environmental factors mostly dietary, gut microbiota disturbances, and host factors.

This review discusses the involvement of the gut microbiota in the pathogenesis of NAFLD, focusing on factors that modulate microbiota composition and intestinal permeability. In addition, NAFLD-associated microbiota signatures, immunological mechanisms behind liver tolerance to gut-derived antigens, and the gut–liver axis will be explained. Finally, we review advances in microbiota-based biomarkers and therapeutic interventions.

## 2. Gut Microbiota Dysbiosis

### 2.1. Introduction to Dysbiosis

Liver diseases, including non-alcoholic fatty liver disease (NAFLD), alcoholic liver disease (ALD), cirrhosis, and hepatocellular carcinoma [9] are associated with compositional and functional alterations of gut microbiota, known as dysbiosis. Dysbiosis is usually characterised by these two major features: (1) the reduction or complete loss of some commensals. The total loss of certain microbial species leads to decreased microbiota diversity, which is associated with many immune-mediated and metabolic disorders [10]. (2) The overgrowth of potentially pathogenic commensals (pathobionts). In a healthy gut ecosystem, pathobionts represent a relatively low percentage of gut microbiota. However, in many diseases, the pathobionts outgrow other commensals. For example, the outgrowth of Gram-negative bacteria from the *Enterobacteriaceae* family, a subgroup of Proteobacteria phylum, is frequently observed in many immune-mediated and metabolic diseases including NAFLD [11,12]. The bloom of Proteobacteria is often considered as a potential diagnostic marker of dysbiosis and risk of disease [13].

Diverse gut microbiota of each individual may endow the host with unique metabolic apparatus and the ability to adapt to changing environment and substrate availability. With decreasing microbial diversity during urbanisation/industrialisation this adaptability was partially lost as human gut microbiota gained new abilities aimed at sugar and xenobiotics processing [14,15].

### 2.2. Triggers and Drivers of Dysbiosis

Dysbiosis might be caused by host-derived factors such as genetic background, health status (infection, inflammation), and lifestyle habits or, even more importantly, by environmental factors such as diet (high in sugars, low in fibre), xenobiotics (antibiotics, medication, food additives, chlorinated water), or hygienic environment.

Profound shifts in gut bacterial and fungal microbiota can be quickly achieved with shifts in macronutrients. These changes have significant physiological consequences, as diets rich in animal protein or simple sugars worsen the intestinal inflammation induced by dextran sulphate sodium. However, while the former increases proinflammatory tuning in gut monocytes the latter worsens the gut barrier function. In both cases, however, interactions between diet and microbiota are necessary for this deleterious effects as they fail to appear in germ-free condition or after transfer of the microbiota to naive mice [16,17].

The effect of food additives on gut microbiota has been long overlooked, but recently, several groups, including ours published data demonstrating that some human gut microbes are highly susceptible to food preservatives [18] and that the exposure to common preservatives promotes Proteobacteria overgrowth [19]. Chassaing et al. found that dietary emulsifiers directly alter human gut microbiota composition, and that the emulsifier-modified microbiota can induce intestinal inflammation when transplanted to germ-free mice [20]. Additionally, Rodriguez-Palacios et al. [21] showed that the artificial sweetener Splenda promotes Proteobacteria dysbiosis and increases myeloperoxidase reactivity in ileitis-prone SAMP mice. In addition, stevioside, another noncaloric sweetener, has been shown to cause structural and functional changes in the gut microbiota [22]. Non-caloric artificial sweeteners have been widely used as sugar substitutes, and their main purpose was to decrease energy intake and prevent the development of obesity and metabolic syndrome. Unfortunately, they often induce dysbiosis and drive glucose intolerance in a microbiota-dependent manner, thus inducing deleterious metabolic effects they were aimed to prevent [23].

Host-derived factors modifying gut microbiota load and composition are bactericidal fluids produced by gastric glands and the liver, i.e., gastric acid and bile, and antimicrobial molecules, such as defensins, lysozymes, and antibacterial lectins (Reg3γ) produced by Paneth cells or SIgA produced by plasma cells [24].

### 2.3. Consequences of Dysbiosis

Dysbiotic microbiota can influence the host immune and metabolic systems and mucosal integrity via various mechanisms. Immune system-modulating mechanisms include the modulation of inflammasome signalling through microbial metabolites, the modulation of Toll-like receptor (TLR) and NOD-like receptor (NLR) signalling, the degradation of secretory IgA (SIgA), the shifting of the balance between regulatory and proinflammatory T cell subsets, direct mucolytic activity and others [25]. Metabolic system-modulating mechanisms include various effects on glucose and lipid metabolism mediated by changes in bile acid composition, the production of short-chain fatty acids (SCFAs) from dietary fibre, the conversion of choline to TMA, and many others [26]. The integrity of the intestinal wall could be disrupted by acetaldehyde produced by microbiota from exogeneous or endogenous ethanol [27], by direct mucolytic activity [28] and other mechanisms.

Whether dysbiosis is a direct cause of NAFLD or merely reflects disease-associated changes in the host’s immune and metabolic systems remains unclear. However, there is accumulating evidence from both preclinical and clinical studies suggesting that gut microbiota dysbiosis plays a key role in the initiation of the disease and its maintenance.

## 3. NAFLD-Associated Microbiota Signatures

### 3.1. Gut Microbiota Signatures

The gut microbiota alterations associated with NAFLD are dependent on the clinical stages of the disease [29]. The most typical general characteristics of the disease’s progression include decreasing microbiota diversity, an increasing abundance of Gram-negative bacteria, mostly Proteobacteria (phylum), and a decreasing abundance of Gram-positive bacteria, mostly Firmicutes (phylum) [30]. Functionally, there is also a shift from beneficial to harmful microbes leading to the development of a proinflammatory and metabolically toxic intestinal environment resulting in gut barrier dysfunction, the exposure of the liver to dietary and microbiota-derived factors, and NAFLD progression [29].

The most consistent gut microbiota signatures associated with NAFLD are increased Proteobacteria (phylum), *Enterobacteriaceae* (family), *Escherichia*, *Bacteroides*, *Dorea*, and *Peptoniphilus* (genus) and decreased *Rikenellaceae*, *Ruminococcaceae* (family), *Faecalibacterium*, *Coprococcus*, *Anaerosporobacter*, and *Eubacterium* (genera) (Table 1). The NAFLD-associated microbiota signatures partially overlap with other metabolic diseases. For example, the levels of *Faecalibacterium prausnitzii* are reduced in cirrhosis [31] as well as in obesity [32], T2DM [33], or in intestinal disorders, such as IBD [34] or IBS [35]. *F. prausnitzii* is considered a beneficial microbe for its anti-inflammatory properties [32]. Another strain, *Bacteroides vulgatus*, which is increased in advanced fibrosis [30], is associated with severe obesity, insulin resistance, and increased haemoglobin A1c levels [36]. Interestingly, cirrhosis patients often have a higher abundance of oral cavity microbial strains, such as *Prevotella*, *Veillonella*, and *Streptococcus* [31], in the gut microbiome, which are generally not present in healthy individuals.

Unlike patients with ALD who have fungal dysbiosis characterised by decreased diversity and *Candida* overgrowth [37], patients with NAFLD have no alterations in gut mycobiome.

**Table 1:** NAFLD-associated gut microbiota signatures.

Phylum	Class	Family	Genus
Proteobacteria↑ [11,12,30,38,39]	Gammaproteobacteria↑ [40]	*Enterobacteriaceae*↑ [11,12]	*Shigella*↑ [11]
			*Escherichia*↑ [12,30,38]
		*Pasteurellaceae*↑ [39]	*Haemophilus*↓ [38]
		*Succinivibrionaceae*↑ [41]	
	Epsilonproteobacteria↑ [40]		
	Alphaproteobacteria	*Kiloniellaceae*↑ [39]	
		*Bradyrhizobiaceae*	*Bradyrhizobium*↑ [42]
Verrucomicrobia↑ [38]	Verrucomicrobiae	*Akkermansiaceae*	*Akkermansia*↑ [38]
Fusobacteria↑ [11]			
Bacteroidetes↑↓ [11,12,40,42,43,44]	Bacteroidia↑ [43]	*Rikenellaceae*↓ [12,42]	*Alistipes*↓ [12]
		*Bacteroidaceae*	*Bacteroides*↑ [45]
	Bacteroidetes	*Prevotellaceae*↑↓ [11,12]	*Prevotella*↑↓ [11,12,40,45]
		*Porphyromonadaceae*↑↓ [39,43]	*Porphyromonas*↑ [12]
			*Parabacteroides*↑ [41]
			*Coprobacter*↓ [38]
Firmicutes↑↓ [12,30,38,39,40,41,42,43]	Clostridia↓ [43]	*Streptococcaceae*↑ [11]	
		*Clostridiaceae*↓ [43]	*Anaerotruncus*↓ [43]
		*Ruminococcaceae*↓ [11,12,39,43]	*Ruminococcus*↑↓ [42,43,45,46,47]
			*Flavonifractor*↑ [38]
			*Subdoligranulum*↓ [38]
			*Faecalibacterium*↓ [12,46,48]
			*Oscillospira*↓ [42]
		*Peptostreptococcaceae*↓ [43]	
		*Lachnospiraceae*↑↓ [11,12,38,39,42,43]	Lachnospiraceae incertae sedis↑ [11]
			*Robinsoniella*↑ [39]
			*Dorea*↑ [39,42]
			*Coprococcus*↓ [12,38,43,46,47]
			*Moryella*↓ [43]
			*Pseudobutyrivibrio*↓ [43]
			*Anaerosporobacter*↓ [43]
			*Roseburia*↑↓ [12,39,43]
			*Blautia*↑↓ [11,12,42,45]
		*Peptoniphilaceae*	*Peptoniphilus*↑ [42,43]
		Clostridiales family XI. incertae sedis	*Anaerococcus*↑ [42]
		*Eubacteriaceae*	*Eubacterium*↓ [12,38]
		*Oscillospiraceae*	*Oscillibacter*↑↓ [38,39,42]
	Negativicutes	*Veillonellaceae*↑ [39]	*Allisonella*↑ [41]
	Erysipelotrichia	*Erysipelotrichaceae*↑ [11]	*Holdemania*↓ [38]
	Bacilli	*Lactobacillaceae*↑↓ [43,46]	*Lactobacillus*↑↓ [39,43,46]
		*Acidaminococcaceae*	*Acidaminococcus*↑ [38]
Actinobacteria↑↓ [12,38,42]	Actinobacteria	*Bifidobacteriaceae*↓ [12]	*Bifidobacterium*↑↓ [12,38]
		*Coriobacteriaceae*	*Eggerthella*↑ [38]

↑ = increased, ↓ = decreased, ↑↓ = contradictory, unassigned = data not available.

### 3.2. Liver and Circulatory Microbiome

Evidence of viable liver or circulating microbiota in NAFLD patients is limited. Nevertheless, bacteria of genera *Staphylococcus* and *Acinetobacter* can be cultured from venous blood of cirrhosis patients [49]. These cirrhosis patients also underwent an implantation of intrahepatic portosystemic shunt (TIPS). During the procedure, portal, hepatic, central, and peripheral venous blood samples were collected and the 16S rRNA analysis showed that the most abundant phylum in all four compartments is Proteobacteria (about 90%) [49].

## 4. Gut–Liver Axis—Bidirectional Link

### 4.1. Definition

**Figure 1:**
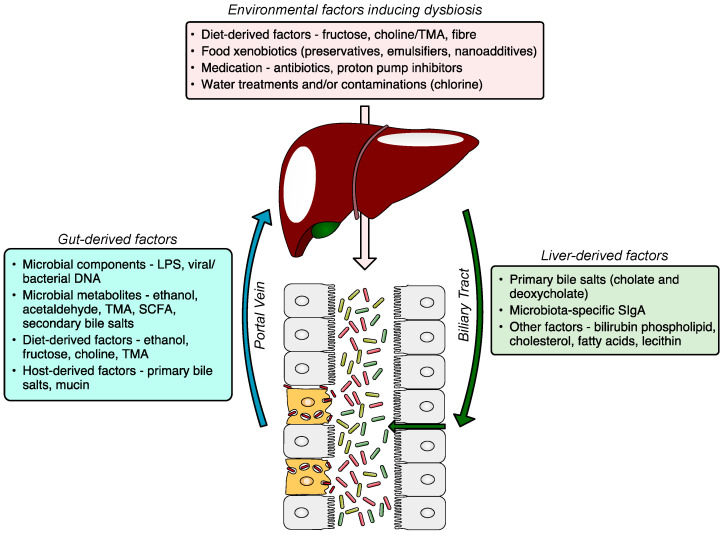
The gut–liver axis and the role of the gut microbiome in the pathogenesis of NAFLD. The gut microbiome altered by environmental factors induces hepatic steatosis. The dysfunctional liver fails to restore gut eubiosis, which results in a vicious self-perpetuating loop, promoting NAFLD progression. The communication via systemic mediators is not shown. NAFLD, non-alcoholic fatty liver disease; MAMP, microbe-associated molecular pattern; LPS, lipopolysaccharide; TMA, trimethylamine; SCFA, short-chain fatty acid; DCA, deoxycholic acid; CDCA, chenodeoxycholic acid.

The gut–liver axis is a bidirectional communication through the biliary tract, portal vein, and systemic circulation. Liver-derived factors, such as bile acids, influence gut microbiota composition and function, and gut-derived products, either dietary or microbial, regulate bile acid synthesis and as well as glucose and lipid metabolism in the liver. The disruption of the gut–liver axis by, for example, environmental factors inducing gut dysbiosis and/or increased intestinal permeability leads to proinflammatory changes in the liver, and its failure to regulate gut microbiota results in further disease progression (Figure 1). A comprehensive understanding of gut–liver communication is a key to developing efficient preventative, diagnostic, and therapeutic approaches.

### 4.2. Intestinal Barrier Dysfunction

The key function of the barrier is to protect tissues and organs from harmful luminal contents, such as parasites, microorganisms, MAMPs, microbial metabolites, dietary antigens, or toxins while preserving nutrient absorption. The intestinal barrier consists of several functional elements. The physical barrier consists of commensal bacteria, mucins secreted by goblet cells, and the intestinal epithelium sealed with tight junction proteins. The immunological barrier includes components of cellular and humoral immunity. Humoral factors, such as antimicrobial peptides and SIgA, control the load and composition of the microbiota in the lumen. The major antimicrobial peptides are defensins, cathelicidins, resistin-like molecules, and lectins produced by specialised epithelial cells and Paneth cells. SIgA is secreted by plasma cells of the lamina propria, activated by antigen-presenting cells, and transported into the intestinal lumen by epithelial cells after binding to the polymeric Ig receptor (pIgR) [50]. SIgA binds to microbial antigens and toxins, protecting the intestinal mucosa from damage, a process known as immune exclusion [51]. The other immunological components that protect the integrity of the mucosa include macrophages and dendritic cells that transport the infiltrated bacteria and antigens to the mesenteric lymph nodes (MLN), allowing the priming and maturation of B and T cells that form the adaptive immune response in the gut-associated lymphoid tissue [52].

There is no consensus as to which factors are major contributors to increased intestinal permeability, however, there is accumulating evidence that environmental factors, especially an unhealthy diet characterised by low fibre, high sugar and HFCS content, and some food additives play a significant role. For example, chronic fructose consumption is associated with tight junction disruption [53] and increased intestinal permeability [54,55]. The excess unabsorbed fructose is metabolised by gut microbiota which results in lactic acidosis. Other factors compromising the intestinal barrier include excessive alcohol consumption, high exposure to medications including antibiotics, stress, and a lack of physical activity. These factors affect gut permeability either directly or via an induction of gut microbiota dysbiosis.

### 4.3. Liver and Immune System

The liver is evolutionarily programmed to tolerate low-level exposure to innocuous dietary and microbial antigens delivered via the portal vein. Liver tolerance is maintained by hepatic antigen-presenting cells (HAPCs), which include dendritic cells, liver sinusoidal endothelial cells (LSECs), Kupffer cells, and hepatic stellate cells [56,57]. Antigen presentation by HAPCs to T cells results in suppression of T cell responses [58]. HAPCs also secrete anti-inflammatory cytokines, such as transforming growth factor-beta (TGF-β) and interleukin 10 (IL-10), in response to low levels of microbiota-derived antigens in portal blood. Both TGF-β and IL-10 promote the differentiation of regulatory T cells (Tregs), which suppress the proliferation and effector functions of CD4+ cells and CD8+ T cells [59,60]. These two immunological mechanisms lead to the induction of liver tolerance and have been shown to protect the liver from immune-mediated liver injury [61,62]. However, when the intestinal barrier is compromised, the liver becomes overloaded with antigens from the gut, leading to a loss of liver tolerance and the development of a proinflammatory milieu. Antigens derived from the microbiota induce inflammation by binding to pattern recognition receptors (PRRs) on liver macrophages, including Kupffer cells and stellate cells [63,64]. Signalling via PRRs, mostly TLRs, leads to increased production of inflammatory (TNFα, IL-1, IL-6) and fibrogenic cytokines/chemokines (TGFβ, MCP-1) as well as oxidative and endoplasmic reticulum (ER) stress [65]. Microbial antigens can also induce type I interferon responses in the liver, leading to proliferation and activation of CD8+ cytotoxic T cells [66]. Other immunological mechanisms, such as various effects of short-chain fatty acids on adaptive immune responses, are discussed separately. All of these immunological mechanisms may contribute to the development of inflammation-mediated liver injury, which may progress to fibrosis, cirrhosis, or even HCC.

## 5. Gut-Derived Factors (Microbial, Dietary, and Host-Derived)

Gut-derived factors involved in pathogenesis of NAFLD might originate in the diet, be a product of gut microbiota, or be host-derived. Dietary factors, such as ethanol, fructose, or choline, might act directly or after processing by microbiota. Microbiota-derived factors comprise microbial components, such as LPS, and products of microbiota metabolism. Host-derived factors are, for example, primary bile salts or mucin.

### 5.1. Microbiota-Derived Components

The exposure of the liver to whole bacteria and/or their components is under normal/healthy conditions insignificant. However, if the gut barrier is disrupted due to the direct effects of dietary factors, such as ethanol or fructose, or indirectly due to gut dysbiosis, the liver is exposed to a significant microbial load. The increased exposure to microorganisms and their products, such as LPS, peptidoglycan, viral or bacterial DNA, or fungal beta-glucan, leads to the induction of proinflammatory changes. These components, collectively labelled microbe-associated molecular patterns (MAMPs), are then recognised by liver innate immune cells (Kupffer cells, dendritic cells, NK and NKT cells, and hepatic stellate cells). The activation and long-term maintenance of inflammation leads to fibrosis, cirrhosis, or even HCC.

The metabolites which are exclusively produced by gut microbiota were identified by comparing metabolomic profiles of germ-free or antibiotic-treated mice with conventional mice. The microbiota-derived metabolites implicated in NAFLD pathogenesis include the choline metabolite trimethylamine (TMA), the secondary bile acids deoxycholic acid (DCA) and lithocholic acid (LCA), and SCFA [67,68,69].

### 5.2. Fructose

Fructose is a monosaccharide that is naturally present in fruits and honey. However, fructose is also a major component of high-fructose corn syrup (HFCS), a ubiquitous sweetener made from corn starch, and sucrose, a glucose-fructose disaccharide. Human exposure to fructose-containing sugars was historically very low. Two hundred years ago, the yearly per capita sugar consumption in Europe and the United States was about 8 kg; by 1900, the consumption had increased to almost 40 kg, and the current consumption of fructose-containing sweeteners including HFCS is close to 70 kg per person per year [70].

Abundant evidence both from preclinical and clinical studies suggests a major role for fructose in the pathogenesis of NAFLD and also NASH [71]. Fructose is known to induce gut microbiota dysbiosis and increase intestinal barrier permeability due to the disruption of tight junctions [53,72]. Other fructose-induced histological changes to the intestinal wall include thinning of the mucosae, loss of crypts and glands, and oedema of the lamina propriae [53,72]. The translocation of microbiota-derived antigens to the liver results in the activation of the innate immune system and hepatic inflammation.

Importantly, fructose also has direct and harmful effects on the liver. The unique metabolism of fructose which is distinct from glucose metabolism leads to ATP depletion [73,74,75], the generation of uric acid [76,77], mitochondrial dysfunctions [73,78], de novo lipogenesis, and the blockage of beta-fatty oxidation [78,79,80]. It is highly likely that chronic exposure to high amounts of fructose in the diet may lead to hepatic fat accumulation or even the development of NASH.

### 5.3. Choline and Its Metabolites

For humans and many animals, choline is an essential nutrient with many functions. It is required for the synthesis of phosphatidylcholine, which in turn is required for the synthesis of cellular membranes and VLDL, which are responsible for the transport of triglycerides out of the liver [81]. Choline is also needed for the production of acetylcholine, the main neurotransmitter of the parasympathetic nervous system. De novo production is possible, but limited, so most of the choline must be obtained from the diet. Choline-rich foods include hen egg yolks, beef liver, meat, and fish. A methionine/choline-deficient diet has been used to induce fatty liver disease in experimental animals for decades [82] and choline supplementation will reverse the disease. Choline deficiency in humans is associated with NAFLD, muscle damage, and neural tube defects [83,84]. Interestingly, 25 years ago Buchman et al. showed that patients on total parenteral nutrition (TPN) develop hepatic steatosis, which could be reversed by intravenous choline supplementation.

However, under certain conditions, choline may play a negative role in NAFLD pathogenesis because specific subsets of gut bacteria can convert it into trimethylamine (TMA) [85,86] which is transported via the portal vein into the liver where is further metabolised to trimethylamine-N-oxide (TMAO) which is perceived as a harmful metabolite [26,87]. The role of gut microbiota in choline metabolism is well documented by the fact that dietary supplementation of choline to germ-free mice does not raise plasma levels of TMA in contrast to conventional mice [68]. Increased serum levels of TMAO are consistently associated with liver steatosis in both preclinical and clinical studies [88].

Choline conversion by microbiota seems to be involved in NAFLD pathogenesis by at least two mechanisms. Firstly, it decreases choline liver bioavailability, which leads to the inefficient export of VLDL particles out of the liver, lipid accumulation, and liver inflammation [89]. Secondly, increased levels of TMAO in the liver promote NAFLD by increasing insulin resistance and decreasing glucose tolerance in mice fed a high-fat diet [90]. Additionally, TMAO, by reducing the activity of CYP7A1 and CYP27A1 enzymes, decreases the conversion of cholesterol into bile acids [91]. Increased levels of TMAO are associated not only with NAFLD, but also with other metabolic diseases, such as atherosclerosis, cardiovascular disease, and type II diabetes [26].

### 5.4. Short Chain Fatty Acids

SCFAs are defined as fatty acids with fewer than six carbons and the most abundant SCFAs are acetate, propionate, and butyrate. They are generated by gut microbiota mainly from dietary nondigestible starch and fibre in the colon and both composition of individual’s microbiota and source of nondigestible fibre determine which SCFAs are produced [92]. They play an important role in many diverse physiological processes, including maintaining gut barrier function, immunomodulation, glucose and lipid metabolism, and appetite regulation. However, increased faecal levels of SCFAs were found to be associated with NAFLD (butyrate and propionate) and liver fibrosis (formate and acetate) [30]. The possible pathogenetic mechanisms include the activation of the G-protein-coupled receptors (GPRs) GPR41 and GPR43, which trigger the release of a gut hormone, peptide YY (PYY), from enteroendocrine cells in the mucosa, which slows intestinal transit time and increases nutrient absorption leading to lipid accumulation in the liver. The influx of SCFAs into the liver via the portal vein triggers gluconeogenesis (propionate) and the accumulation of triglycerides (acetate) both of which are associated with NAFLD [93].

On the other hand, butyrate is a major source of energy for colonocytes, and the supplementation of tributyrin, a butyrate prodrug, as well as acetate and propionate, was shown to protect against diet-induced obesity, hepatic steatosis, and insulin resistance [94,95].

Published data on the immunomodulatory effects of SCFAs are mostly positive, i.e., favouring the induction of anti-inflammatory T cells. However, the evidence that SCFAs might, under certain conditions, induce proinflammatory T cells, such as Th1 and Th17 exists [96,97]. Interestingly, systemic acetate improves rapid recall response in memory CD8+ T cells, which allow better control of the infection by intracellular parasites [98].

Obviously, further research is needed to determine how significant the contribution of SCFA in NAFLD pathogenesis is and to discriminate whether the net effect is positive or negative. Additionally, it is important to discriminate between circulating and faecal SCFA levels as circulating SCFA are more directly linked to metabolic health [99].

### 5.5. Ethanol and its Metabolites

Even healthy subjects with no alcohol consumption have low levels of blood ethanol [100], and gut microbiota is one of the sources of endogenous ethanol. Yuan et al. recently showed that some human intestinal strains, such as *Klebsiella pneumoniae*, are significant alcohol producers and that the NAFLD phenotype is transferable to experimental mice using FMT [101].

NAFLD has been associated with increased luminal and serum levels of ethanol and its metabolites, acetate, and acetaldehyde. Additionally, children with an inflammatory form of NAFLD, NASH, have increased serum levels of ethanol compared to healthy children [12]. However, the significance of endogenous ethanol production in NAFLD pathogenesis is not clear, and further research is needed.

Increased levels of endogenous ethanol and its metabolites fuel the progression of the disease by several mechanisms. In the gut, ethanol and its metabolite, acetaldehyde, increase intestinal permeability by stimulating the production of inflammatory cytokines, decreasing the production of AMPs, and disrupting tight junctions. This leads to gut barrier dysfunction, the translocation of microbiota-derived molecules into the liver resulting in increased production of inflammatory cytokines and the induction of lipogenesis. In the liver, ethanol disrupts lipid metabolism by inducing fatty acid uptake and de novo lipogenesis, impairing fatty acid oxidation and inhibiting VLDL export [102]. Continuous exposure to ethanol also increases the activity of the ethanol oxidising enzyme CYP2E1 [103], which leads to an increased production of free radicals, which together with increased levels of acetaldehyde and acetate further promote liver damage.

Interestingly, gut microbiota can also metabolise ethanol and protect the liver against alcohol-induced liver injury. This was well documented by a study in germ-free mice showing that the absence of microbiota leads to increased liver exposure to ethanol, increased expression of ethanol metabolising enzymes, and exacerbation of hepatic steatosis [104]. Therefore, the gut microbiota plays an essential role in ethanol metabolism as it can either increase or decrease the host’s exposure to ethanol depending on its compositional and functional setting.

## 6. Liver-Derived Factors

### 6.1. Bile Acids and Their Metabolites

The primary bile acids (BAs), i.e., cholic and chenodeoxycholic (CDCA), are organic molecules synthesised in a multistep process from cholesterol in the pericentral hepatocytes and secreted in the form of glycine/taurine conjugated bile salts via the bile duct together with other bile components such as bilirubin phospholipid, cholesterol, amino acids, porphyrins, and xenobiotics, into the duodenum [105]. The BAs are amphipathic molecules, i.e., having both hydrophilic and hydrophobic parts, making them excellent emulsifiers. Therefore, the main function of BAs and their salts is to facilitate digestion and the absorption of lipids and lipid-soluble vitamins. The relation with gut microbiota is bidirectional. The BAs prevent the overgrowth of bacteria in the small intestine (SIBO) and control the composition of the microbiota, and on the other hand, the microbiota can metabolise bile salts back into primary BAs or further into secondary BAs, i.e., deoxycholic (DCA) and lithocholic. The 95% of BAs and their salts is actively absorbed in the ileum, transported via the portal vein back into the liver, and recycled. The remainder is passively absorbed in the colon or excreted. The changes in BA secretion may be responsible for reproducible alterations of gut microbial communities during dietary shifts [106].

Several preclinical and clinical studies associate the disturbance of bile acid metabolism with NAFLD. There are two well-studied mechanistic pathways implicated in the NAFLD pathogenesis. Firstly, a decreased signalling via the bile acid receptor farnesoid X receptor (FXR) due to an increased ratio of secondary to primary BAs (DCA/CDCA) leads to dysregulation of lipid and glucose metabolism [107,108]. Additionally this decreased FXR activation leads to lower levels of fibroblast growth factor 19 (FGF19) and the inhibition of cholesterol 7α-monooxygenase (CYP7A1), which is a key BA synthesis controlling enzyme. It is conceivable that gut microbiota dysbiosis will modify the balance between primary and secondary BAs and following disruption of FXR signalling will have far-reaching effects on host metabolism. Secondly, BAs by binding to the G-protein-coupled receptor (TGR5) in the intestine and liver can regulate glucose homeostasis and suppress inflammatory cytokines production by Kupffer cells via the TGR5-cAMP pathway [109]. Several FXR and TGR5 agonists are being tested in clinical trials with positive results. For example, obeticholic acid, a potent activator of the FXR receptor, improves the histological features of NASH [110].

### 6.2. Immunoglobulin A

The primary source of SIgA in the intestine is intestinal plasma cells. However, plasma cells capable of producing microbiota-specific SIgA are also present in the liver [111]. The liver SIgA is transported via pIgR expressed on biliary epithelial cells into bile [112]. This mechanism is similar to the SIgA production in mammary glands. Maternal milk containing SIgA plays an important role in establishing a healthy gut microbiome in nursing infants [113]. However, the effects of the liver-produced biliary SIgA on intestinal microbiota and its significance are not well understood.

## 7. Conventional Noninvasive Diagnostic and Prognostic Methods

The most common diagnostic and prognostic methods for NAFLD are blood tests, imaging methods, and liver stiffness assessment methods. Blood tests are used to detect elevated liver enzymes such as alanine aminotransferase (ALT) and aspartate aminotransferase (AST). Such laboratory abnormalities are often the first sign of liver dysfunction. The most common tests used to visualise the liver include ultrasound, computed tomography (CT), and magnetic resonance imaging (MRI). MRI is the most sensitive test for imaging hepatic steatosis and can be used to calculate the amount of fat in the liver. Liver stiffness or elasticity could be measured with ultrasound or magnetic resonance elastography (MRE). The low frequency vibrations are visualised to create a map, an elastogram, that shows the stiffness and elasticity over the liver. Elastography methods can detect the early stages of liver fibrosis and may eliminate the need for an invasive test, i.e., liver biopsy [114,115].

## 8. Microbiota-Based Biomarkers and Therapeutic Interventions

An increased knowledge of gut microbiota, MAMPs, and microbiota-derived metabolites’ involvement in the NAFLD pathogenesis could be exploited in novel diagnostic and therapeutic approaches. NAFLD is a heterogeneous group of conditions, which consist of several subtypes driven by various combinations of the above-mentioned factors, and this knowledge should be reflected in both diagnosis and therapy.

### 8.1. Microbiota-Based Diagnostics and Biomarkers

Currently, a liver biopsy is still required for the diagnosis and monitoring of disease progression. Therefore, noninvasive and reliable markers for NAFLD evaluation are urgently needed. Recent progress in gut microbiota research suggests that some microbiota members and metabolites might be useful as diagnostic and prognostic markers. For example, Loomba et al. found that a panel of gut microbiota consisting of 37 bacterial strains can be used to accurately diagnose advanced fibrosis in patients with NAFLD [30] and Lee et al. have found a strong association of *Veillonellaceae* with liver fibrosis in non-obese NAFLD patients and suggest *Veillonellaceae* as a diagnostic marker [116].

There are several microbiota-derived metabolites that could be used as NAFLD biomarkers. Some of the most promising are succinate, phenylacetic acid, and 3-(4-hydroxyphenyl) lactate. Interestingly, NAFLD patients have often a decreased microbial gene richness resulting in an alteration in aromatic amino acid and branched-chain amino acid metabolism. For example, the above mentioned 3-(4-hydroxyphenyl) lactate which is associated with liver fibrosis, is a product of aromatic amino acid metabolism [117]. Serum levels of phenylacetic acid correlate with the severity of liver steatosis [38]. Succinate, which is produced by NAFLD-associated microbes such as *Bacteroidaceae* and *Prevotella* [45], was found to be increased in faecal, serum, and liver samples of NAFLD patients [30].

### 8.2. Microbiota-Based Therapies

The deep knowledge of gut microbiota could be exploited for therapeutic purposes on several levels. Firstly, the whole microbiota communities might be restored using FMT. Secondly, single strains or collections of beneficial strains (probiotics) could be introduced to gut microbiota to deliver missing functionality while harmful or undesirable strains might be deleted using antibiotics, antimycotics, or bacteriophages. Lastly, microbial metabolic pathways could be targeted to decrease or block the production of harmful metabolites or to stimulate the production of those which are beneficial.

Data on the efficacy of FMT in the treatment of NAFLD are limited. To date, FMT has been used successfully in cirrhotic patients with hepatic encephalopathy [118] and alcoholic hepatitis [119].

Probiotics, prebiotics, and synbiotics have also been used in NAFLD treatment. Probiotics are nonpathogenic live microorganisms that, when consumed, can have a positive effect on host health [120]. The most commonly used probiotics in clinical trials are lactobacilli, streptococci, and bifidobacteria. Prebiotics were originally defined as indigestible dietary components that selectively stimulate the growth and activity of beneficial gut bacteria [121]. However, this definition was later expanded to include not only fibre-based probiotics but also other substrates that are selectively utilised by the host microbiota and provide health benefits. The new definition includes not only indigestible carbohydrates such as fructo-oligosaccharides (FOS), galacto-oligosaccharides (GOS) and trans-galacto-oligosaccharides (TOS), but also other substances such as polyunsaturated fatty acids (PUFAs) and polyphenols [122] that have the ability to modulate the gut microbiota [123]. Synbiotics are a mixture of probiotics and prebiotics that have beneficial effects on the host. Data from both animal and clinical studies suggest that probiotics/synbiotics have the potential to ameliorate NAFLD-associated dysbiosis and liver pathology. For example, a recent meta-analysis [124] found that the use of probiotics/synbiotics was associated with improvement in liver-specific markers of hepatic inflammation, liver stiffness, and steatosis in NAFLD patients.

The therapeutic strategy of targeting single strain, specifically cytolytic *E. faecalis*, using a bacteriophage was shown to be effective in ethanol-induced liver disease in humanised mice [125].

The therapeutic approaches aimed at manipulating microbiota to increase the production of protective metabolites or to block/decrease the production of harmful metabolites are also promising. For example, the conversion of dietary choline into TMA by microbial TMA lyases can be blocked by 3,3-dimethyl-1-butanol, which is a structural analogue of choline [126]. Microbiota manipulation to increase SCFA production might also be helpful as preclinical data show that SCFA supplementation improves liver steatosis. For example, the supplementation of tributyrin, a butyrate prodrug, protected mice against obesity, insulin resistance, and hepatic steatosis [94] while the supplementation of acetate and propionate prevents diet-induced weight gain, hepatic steatosis, and insulin resistance [95]. Other promising therapeutic targets are FXR and TGR5 signalling pathways modulating bile acid metabolism. For example, obeticholic acid, which is a potent activator of FXR, improves hepatic steatosis, fibrosis, and portal hypertension in animal models of fatty liver disease [127,128,129] and the treatment of adult NASH patients resulted in improved histological features [110]. Additionally, NGM282, an analogue of FGF19, i.e., a hormone regulating bile acid synthesis and glucose homeostasis, was shown to reduce hepatic steatosis in NASH patients [130].

## 9. Conclusions

Increasing evidence suggests that the gut microbiota plays a critical role in the pathogenesis of NAFLD. Metabolites derived from the gut, whether beneficial or harmful, are transported via the portal vein directly to the liver for further processing, including energy production, detoxification, and synthesis. Problems arise when the liver becomes overloaded with harmful, toxic or proinflammatory molecules due to unhealthy dietary habits, dysbiosis of the gut microbiota and/or increased gut permeability. These molecules originate from either the diet (fructose, ethanol, additives, antibiotics) or the microbiota (LPS, bacterial/viral DNA, TMA, secondary bile acids, ethanol, or acetaldehyde) and negatively affect liver physiology. The dysfunctional liver cannot effectively control the gut microbiota via bile acids and other microbiome-regulating factors, resulting in gut dysbiosis and gut barrier dysfunction. This reinforcing loop has deleterious effects on the liver, gut, and human health in general.

The major disruptors of the gut microbiota are environmental factors, particularly diet and medications. Genetic factors provide the playing field for NAFLD pathogenesis but may not be responsible for the steady increase in NAFLD incidence due to their relatively stable nature.

Several clinical studies have uncovered promising NAFLD-associated microbiome signatures that could be used for noninvasive NAFLD diagnosis or monitoring of disease progression. However, the usefulness of the signatures needs to be further validated in well-designed large cohort longitudinal studies that take into account important confounding factors such as comorbidities, including T2DM and obesity, ethnic background, medication and diet. The diagnostic and prognostic value of microbiota signatures could be further enhanced by combining them with microbiota-derived metabolites detected in blood/plasma, urine, or faeces. Such an approach will allow more accurate differentiation of NAFLD subtypes and more precise evaluation of the efficacy of therapy.

A comprehensive understanding of the interactions between microbiota and liver will enable us to develop effective microbiota-based therapies. There will be two main approaches that can be used in combination. The first will be direct manipulation of the gut microbiota by removing unwanted strains, introducing beneficial strains (preferably isolated from the healthy human gut microbiota) or replacing the dysbiotic microbiota with FMT. The second approach will be based on exploiting microbial metabolites by either inducing or blocking their production.

In summary, a growing body of evidence suggests that NAFLD is triggered and driven by a gut microbiota distorted by environmental factors. A deep knowledge of the interactions between microbiota and liver will allow us to introduce new noninvasive diagnostic and prognostic methods based on NAFLD-specific microbiota signatures and metabolites, and also to develop effective microbiota-based therapies, such as probiotic interventions, FMT, or metabolite manipulations.

## References

[1] European Association for the Study of the Liver (EASL), European Association for the Study of Diabetes (EASD), European Association for the Study of Obesity (EASO) (2016). EASL-EASD-EASO Clinical Practice Guidelines for the Management of Non-Alcoholic Fatty Liver Disease. Obes. Facts.

[2] Bajaj J.S. (2019). Alcohol, liver disease and the gut microbiota. Nat. Rev. Gastroenterol. Hepatol..

[3] Ballestri S., Nascimbeni F., Romagnoli D., Lonardo A. (2016). The independent predictors of non-alcoholic steatohepatitis and its individual histological features.: Insulin resistance, serum uric acid, metabolic syndrome, alanine aminotransferase and serum total cholesterol are a clue to pathogenesis and candidate targets for treatment. Hepatol. Res..

[4] Stepanova M., Rafiq N., Makhlouf H., Agrawal R., Kaur I., Younoszai Z., McCullough A., Goodman Z., Younossi Z.M. (2013). Predictors of All-Cause Mortality and Liver-Related Mortality in Patients with Non-Alcoholic Fatty Liver Disease (NAFLD). Dig. Dis. Sci..

[5] Vanni E., Bugianesi E., Kotronen A., De Minicis S., Yki-Järvinen H., Svegliati-Baroni G. (2010). From the metabolic syndrome to NAFLD or vice versa?. Dig. Liver Dis..

[6] Athyros V.G., Alexandrides T.K., Karagiannis A., Karvounis C., Katsiki N., Kotsis V., Kountouras J., Liberopoulos E., Pitsavos C., Polyzos S. (2017). The use of statins alone, or in combination with pioglitazone and other drugs, for the treatment of non-alcoholic fatty liver disease/non-alcoholic steatohepatitis and related cardiovascular risk. An Expert Panel Statement. Metabolism.

[7] Younossi Z.M., Koenig A.B., Abdelatif D., Fazel Y., Henry L., Wymer M. (2016). Global epidemiology of nonalcoholic fatty liver disease-Meta-analytic assessment of prevalence, incidence, and outcomes. Hepatology.

[8] Younossi Z., Anstee Q.M., Marietti M., Hardy T., Henry L., Eslam M., George J., Bugianesi E. (2018). Global burden of NAFLD and NASH: Trends, predictions, risk factors and prevention. Nat. Rev. Gastroenterol. Hepatol..

[9] Betrapally N.S., Gillevet P.M., Bajaj J.S. (2017). Gut microbiome and liver disease. Transl. Res..

[10] Carding S., Verbeke K., Vipond D.T., Corfe B.M., Owen L.J. (2015). Dysbiosis of the gut microbiota in disease. Microb. Ecol. Health Dis..

[11] Shen F., Zheng R.-D., Sun X.-Q., Ding W.-J., Wang X.-Y., Fan J.-G. (2017). Gut microbiota dysbiosis in patients with non-alcoholic fatty liver disease. Hepatobiliary Pancreat. Dis. Int..

[12] Zhu L., Baker S.S., Gill C., Liu W., Alkhouri R., Baker R.D., Gill S.R. (2013). Characterization of gut microbiomes in nonalcoholic steatohepatitis (NASH) patients: A connection between endogenous alcohol and NASH. Hepatology.

[13] Shin N.-R., Whon T.W., Bae J.-W. (2015). Proteobacteria: Microbial signature of dysbiosis in gut microbiota. Trends Biotechnol..

[14] Gomez A., Petrzelkova K.J., Burns M.B., Yeoman C.J., Amato K.R., Vlckova K., Modry D., Todd A., Robinson C.A.J., Remis M.J. (2016). Gut Microbiome of Coexisting BaAka Pygmies and Bantu Reflects Gradients of Traditional Subsistence Patterns. Cell Rep..

[15] Mancabelli L., Milani C., Lugli G.A., Turroni F., Ferrario C., Van Sinderen D., Ventura M. (2017). Meta-analysis of the human gut microbiome from urbanized and pre-agricultural populations. Environ. Microbiol..

[16] Kostovcikova K., Coufal S., Galanova N., Fajstova A., Hudcovic T., Kostovcik M., Prochazkova P., Zakostelska Z.J., Cermakova M., Sediva B. (2019). Diet Rich in Animal Protein Promotes Pro-inflammatory Macrophage Response and Exacerbates Colitis in Mice. Front. Immunol..

[17] Fajstova A., Galanova N., Coufal S., Malkova J., Kostovcik M., Cermakova M., Pelantova H., Kuzma M., Sediva B., Hudcovic T. (2020). Diet Rich in Simple Sugars Promotes Pro-Inflammatory Response via Gut Microbiota Alteration and TLR4 Signaling. Cells.

[18] Hrncirova L., Machova V., Trckova E., Krejsek J., Hrncir T. (2019). Food Preservatives Induce Proteobacteria Dysbiosis in Human-Microbiota Associated Nod2-Deficient Mice. Microorganisms.

[19] Hrncirova L., Hudcovic T., Sukova E., Machova V., Trckova E., Krejsek J., Hrncir T. (2019). Human gut microbes are susceptible to antimicrobial food additives in vitro. Folia Microbiol..

[20] Chassaing B., Van De Wiele T., De Bodt J., Marzorati M., Gewirtz A.T. (2017). Dietary emulsifiers directly alter human microbiota composition and gene expression ex vivo potentiating intestinal inflammation. Gut.

[21] Rodriguez-Palacios A., Harding A., Menghini P., Himmelman C., Retuerto M., Nickerson K.P., Lam M., Croniger C.M., McLean M.H., Durum S.K. (2018). The Artificial Sweetener Splenda Promotes Gut Proteobacteria, Dysbiosis, and Myeloperoxidase Reactivity in Crohn’s Disease–Like Ileitis. Inflamm. Bowel Dis..

[22] Gatea F., Sârbu I., Vamanu E. (2021). In Vitro Modulatory Effect of Stevioside, as a Partial Sugar Replacer in Sweeteners, on Human Child Microbiota. Microorganisms.

[23] Suez J., Korem T., Kuperman Y., Harmelin A., Kolodkin-Gal I., Shapiro H., Halpern Z., Segal E., Elinav E., Zeevi D. (2014). Artificial sweeteners induce glucose intolerance by altering the gut microbiota. Nature.

[24] Catanzaro J.R., Strauss J.D., Bielecka A., Porto A.F., Lobo F.M., Urban A., Schofield W.B., Palm N.W. (2019). IgA-deficient humans exhibit gut microbiota dysbiosis despite secretion of compensatory IgM. Sci. Rep..

[25] Levy M., Kolodziejczyk A.A., Thaiss C.A., Elinav E. (2017). Dysbiosis and the immune system. Nat. Rev. Immunol..

[26] Chu H., Duan Y., Yang L., Schnabl B. (2018). Small metabolites, possible big changes: A microbiota-centered view of non-alcoholic fatty liver disease. Gut.

[27] Llorente C., Schnabl B. (2015). The Gut Microbiota and Liver Disease. Cell. Mol. Gastroenterol. Hepatol..

[28] Png C.W., Lindén S.K., Gilshenan K.S., Zoetendal E.G., McSweeney C.S., Sly L.I., McGuckin M.A., Florin T.H.J. (2010). Mucolytic Bacteria with Increased Prevalence in IBD Mucosa Augment In Vitro Utilization of Mucin by Other Bacteria. Am. J. Gastroenterol..

[29] Aron-Wisnewsky J., Vigliotti C., Witjes J., Le P., Holleboom A.G., Verheij J., Nieuwdorp M., Clément K. (2020). Gut microbiota and human NAFLD: Disentangling microbial signatures from metabolic disorders. Nat. Rev. Gastroenterol. Hepatol..

[30] Loomba R., Seguritan V., Li W., Long T., Klitgord N., Bhatt A., Dulai P.S., Caussy C., Bettencourt R., Highlander S.K. (2017). Gut Microbiome-Based Metagenomic Signature for Non-invasive Detection of Advanced Fibrosis in Human Nonalcoholic Fatty Liver Disease. Cell Metab..

[31] Qin N., Yang F., Li A., Prifti E., Chen Y., Shao L., Guo J., Le Chatelier E., Yao J., Wu L. (2014). Alterations of the human gut microbiome in liver cirrhosis. Nature.

[32] Le Chatelier E., Nielsen T., Qin J., Prifti E., Hildebrand F., Falony G., Almeida M., Arumugam M., Batto J.-M., Kennedy S. (2013). Richness of human gut microbiome correlates with metabolic markers. Nature.

[33] Karlsson F.H., Tremaroli V., Nookaew I., Bergström G., Behre C.J., Fagerberg B., Nielsen J., Bäckhed F. (2013). Gut metagenome in European women with normal, impaired and diabetic glucose control. Nature.

[34] Sokol H., Seksik P., Furet J.P., Firmesse O., Nion-Larmurier I., Beaugerie L., Cosnes J., Corthier G., Marteau P., Doré J. (2009). Low counts of Faecalibacterium prausnitzii in colitis microbiota. Inflamm. Bowel Dis..

[35] Rajilić–Stojanović M., Biagi E., Heilig H.G., Kajander K., Kekkonen R.A., Tims S., de Vos W.M. (2011). Global and Deep Molecular Analysis of Microbiota Signatures in Fecal Samples From Patients with Irritable Bowel Syndrome. Gastroenterology.

[36] Aron-Wisnewsky J., Prifti E., Belda E., Ichou F., Kayser B.D., Dao M.C., Verger E.O., Hedjazil L., Bouillot J.-L., Chevallier J.-M. (2019). Major microbiota dysbiosis in severe obesity: Fate after bariatric surgery. Gut.

[37] Yang A.-M., Inamine T., Hochrath K., Chen P., Wang L., Llorente C., Bluemel S., Hartmann P., Xu J., Koyama Y. (2017). Intestinal fungi contribute to development of alcoholic liver disease. J. Clin. Investig..

[38] Hoyles L., Fernandez-Real J.-M., Federici M., Serino M., Abbott J., Charpentier J., Heymes C., Luque J.L., Anthony E., Barton R.H. (2018). Molecular phenomics and metagenomics of hepatic steatosis in non-diabetic obese women. Nat. Med..

[39] Raman M., Ahmed I., Gillevet P.M., Probert C.S., Ratcliffe N.M., Smith S., Greenwood R., Sikaroodi M., Lam V., Crotty P. (2013). Fecal Microbiome and Volatile Organic Compound Metabolome in Obese Humans with Nonalcoholic Fatty Liver Disease. Clin. Gastroenterol. Hepatol..

[40] Michail S., Lin M., Frey M.R., Fanter R., Paliy O., Hilbush B., Reo N.V. (2014). Altered gut microbial energy and metabolism in children with non-alcoholic fatty liver disease. FEMS Microbiol. Ecol..

[41] Wong V.W.-S., Tse C.-H., Lam T.T.-Y., Wong G.L.-H., Chim A.M.-L., Chu W.C.-W., Yeung D.K.-W., Law P.T.-W., Kwan H.-S., Yu J. (2013). Molecular Characterization of the Fecal Microbiota in Patients with Nonalcoholic Steatohepatitis–A Longitudinal Study. PLoS ONE.

[42] Del Chierico F., Nobili V., Vernocchi P., Russo A., De Stefanis C., Gnani D., Furlanello C., Zandonà A., Paci P., Capuani G. (2017). Gut microbiota profiling of pediatric nonalcoholic fatty liver disease and obese patients unveiled by an integrated meta-omics-based approach. Hepatology.

[43] Wang B., Jiang X., Cao M., Ge J., Bao Q., Tang L., Chen Y., Li L. (2016). Altered Fecal Microbiota Correlates with Liver Biochemistry in Nonobese Patients with Non-alcoholic Fatty Liver Disease. Sci. Rep..

[44] Mouzaki M., Comelli E.M., Arendt B.M., Bonengel J., Fung S.K., Fischer S.E., McGilvray I.D., Allard J.P. (2013). Intestinal microbiota in patients with nonalcoholic fatty liver disease. Hepatology.

[45] Boursier J., Mueller O., Hunault G., Oberti F., Calès P., Diehl A.M., Barret M., Machado M.V., Fizanne L., Araujo-Perez F. (2016). The severity of nonalcoholic fatty liver disease is associated with gut dysbiosis and shift in the metabolic function of the gut microbiota. Hepatology.

[46] Da Silva H.E., Teterina A., Comelli E.M., Taibi A., Arendt B.M., Fischer S.E., Lou W., Allard J.P. (2018). Nonalcoholic fatty liver disease is associated with dysbiosis independent of body mass index and insulin resistance. Sci. Rep..

[47] Alferink L.J., Radjabzadeh D., Erler N.S., Vojinovic D., Medina-Gomez C., Uitterlinden A.G., de Knegt R.J., Amin N., Ikram M.A., Janssen H.L. (2021). Microbiomics, Metabolomics, Predicted Metagenomics, and Hepatic Steatosis in a Population-Based Study of 1355 Adults. Hepatology.

[48] Chen Y., Yang F., Lu H., Wang B., Chen Y., Lei D., Wang Y., Zhu B., Li L. (2011). Characterization of fecal microbial communities in patients with liver cirrhosis. Hepatology.

[49] Schierwagen R., Alvarez-Silva C., Madsen M.S.A., Kolbe C.C., Meyer C., Thomas D., Uschner F.E., Magdaleno F., Jansen C., Pohlmann A. (2019). Circulating microbiome in blood of different circulatory compartments. Gut.

[50] Brandtzaeg P., Prydz H. (1984). Direct evidence for an integrated function of J chain and secretory component in epithelial transport of immunoglobulins. Nature.

[51] Mestecky J., Russell M.W., Elson C.O. (1999). Intestinal IgA: Novel views on its function in the defence of the largest mucosal surface. Gut.

[52] Gautreaux M.D., Deitch E.A., Berg R.D. (1994). T lymphocytes in host defense against bacterial translocation from the gastrointestinal tract. Infect. Immun..

[53] Johnson R.J., Rivard C., Lanaspa M.A., Otabachian-Smith S., Ishimoto T., Cicerchi C., Cheeke P.R., MacIntosh B., Hess T. (2013). Fructokinase, Fructans, Intestinal Permeability, and Metabolic Syndrome: An Equine Connection?. J. Equine Veter. Sci..

[54] Spruss A., Bergheim I. (2009). Dietary fructose and intestinal barrier: Potential risk factor in the pathogenesis of nonalcoholic fatty liver disease. J. Nutr. Biochem..

[55] Bergheim I., Weber S., Vos M., Krämer S., Volynets V., Kaserouni S., McClain C.J., Bischoff S.C. (2008). Antibiotics protect against fructose-induced hepatic lipid accumulation in mice: Role of endotoxin. J. Hepatol..

[56] Crispe I.N. (2011). Liver antigen-presenting cells. J. Hepatol..

[57] Horst A.K., Neumann K., Diehl L., Tiegs G. (2016). Modulation of liver tolerance by conventional and nonconventional antigen-presenting cells and regulatory immune cells. Cell. Mol. Immunol..

[58] Karimi M.H., Geramizadeh B., Malek-Hosseini S.A. (2015). Tolerance Induction in Liver. Int. J. Organ Transplant. Med..

[59] Breous E., Somanathan S., Vandenberghe L.H., Wilson J.M. (2009). Hepatic regulatory T cells and Kupffer cells are crucial mediators of systemic T cell tolerance to antigens targeting murine liver. Hepatology.

[60] Carambia A., Freund B., Schwinge D., Heine M., Laschtowitz A., Huber S., Wraith D.C., Korn T., Schramm C., Lohse A.W. (2014). TGF-β-dependent induction of CD4+CD25+Foxp3+ Tregs by liver sinusoidal endothelial cells. J. Hepatol..

[61] Crispe I.N. (2014). Immune tolerance in liver disease. Hepatology.

[62] Doherty D.G. (2019). Antigen-specific immune tolerance in the liver. Nat. Biomed. Eng..

[63] Isayama F., Hines I.N., Kremer M., Milton R.J., Byrd C.L., Perry A.W., McKim S.E., Parsons C., Rippe R.A., Wheeler M.D. (2006). LPS signaling enhances hepatic fibrogenesis caused by experimental cholestasis in mice. Am. J. Physiol. Gastrointest. Liver Physiol..

[64] Gäbele E., Mühlbauer M., Dorn C., Weiss T.S., Froh M., Schnabl B., Wiest R., Schölmerich J., Obermeier F., Hellerbrand C. (2008). Role of TLR9 in hepatic stellate cells and experimental liver fibrosis. Biochem. Biophys. Res. Commun..

[65] Lebeaupin C., Proics E., De Bieville C.H.D., Rousseau D., Bonnafous S., Patouraux S., Adam G., Lavallard V.J., Rovere C., Le Thuc O. (2015). ER stress induces NLRP3 inflammasome activation and hepatocyte death. Cell Death Dis..

[66] Ghazarian M., Revelo X.S., Nøhr M.K., Luck H., Zeng K., Lei H., Tsai S., Schroer S.A., Park Y.J., Chng M.H.Y. (2017). Type I interferon responses drive intrahepatic T cells to promote metabolic syndrome. Sci. Immunol..

[67] Studer N., Desharnais L., Beutler M., Brugiroux S., Terrazos M.A., Menin L., Schürch C.M., McCoy K.D., Kuehne S.A., Minton N.P. (2016). Functional Intestinal Bile Acid 7α-Dehydroxylation by Clostridium scindens Associated with Protection from Clostridium difficile Infection in a Gnotobiotic Mouse Model. Front. Cell. Infect. Microbiol..

[68] Zhu W., Gregory J.C., Org E., Buffa J.A., Gupta N., Wang Z., Li L., Fu X., Wu Y., Mehrabian M. (2016). Gut Microbial Metabolite TMAO Enhances Platelet Hyperreactivity and Thrombosis Risk. Cell.

[69] Yajima M., Karaki S.-I., Tsuruta T., Kimura S., Nio-Kobayashi J., Kuwahara A., Yajima T. (2016). Diversity of the intestinal microbiota differently affects non-neuronal and atropine-sensitive ileal contractile responses to short-chain fatty acids in mice. Biomed. Res..

[70] Johnson R.J., Segal M.S., Sautin Y., Nakagawa T., Feig D.I., Kang D.-H., Gersch M.S., Benner S., Sánchez-Lozada L.G. (2007). Potential role of sugar (fructose) in the epidemic of hypertension, obesity and the metabolic syndrome, diabetes, kidney disease, and cardiovascular disease. Am. J. Clin. Nutr..

[71] Jensen T., Abdelmalek M.F., Sullivan S., Nadeau K.J., Green M., Roncal C., Nakagawa T., Kuwabara M., Sato Y., Kang D.-H. (2018). Fructose and sugar: A major mediator of non-alcoholic fatty liver disease. J. Hepatol..

[72] Li J.-M., Yu R., Zhang L.-P., Wen S.-Y., Wang S.-J., Zhang X.-Y., Xu Q., Kong L.-D. (2019). Dietary fructose-induced gut dysbiosis promotes mouse hippocampal neuroinflammation: A benefit of short-chain fatty acids. Microbiome.

[73] Mäenpää P.H., Raivio K.O., Kekomäki M.P. (1968). Liver Adenine Nuldeotides: Fructose-Induced Depletion and Its Effect on Protein Synthesis. Science.

[74] Abdelmalek M.F., Lazo M., Horska A., Bonekamp S., Lipkin E.W., Balasubramanyam A., Bantle J.P., Johnson R.J., Diehl A.M., The Fatty Liver Subgroup of the Look AHEAD Research Group (2012). Higher dietary fructose is associated with impaired hepatic adenosine triphosphate homeostasis in obese individuals with type 2 diabetes. Hepatology.

[75] Bawden S., Stephenson M., Ciampi E., Hunter K., Marciani L., Macdonald I., Aithal G., Morris P., Gowland P. (2016). Investigating the effects of an oral fructose challenge on hepatic ATP reserves in healthy volunteers: A 31P MRS study. Clin. Nutr..

[76] Berghe G.V.D. (1986). Fructose: Metabolism and short-term effects on carbohydrate and purine metabolic pathways. Prog. Biochem. Pharmacol..

[77] Le M.T., Frye R.F., Rivard C.J., Cheng J., McFann K.K., Segal M.S., Johnson R.J., Johnson J.A. (2012). Effects of high-fructose corn syrup and sucrose on the pharmacokinetics of fructose and acute metabolic and hemodynamic responses in healthy subjects. Metabolism.

[78] Lanaspa M.A., Sanchez-Lozada L.G., Choi Y.-J., Cicerchi C., Kanbay M., Roncal-Jimenez C.A., Ishimoto T., Li N., Marek G., Duranay M. (2012). Uric Acid Induces Hepatic Steatosis by Generation of Mitochondrial Oxidative Stress: Potential Role in Fructose-Dependent and- Independent Fatty Liver. J. Biol. Chem..

[79] Lim J.S., Mietus-Snyder M., Valente A., Schwarz J.-M., Lustig R.H. (2010). The role of fructose in the pathogenesis of NAFLD and the metabolic syndrome. Nat. Rev. Gastroenterol. Hepatol..

[80] Lanaspa M.A., Cicerchi C., Garcia G., Li N., Roncal-Jimenez C.A., Rivard C.J., Hunter B., Andrés-Hernando A., Ishimoto T., Sánchez-Lozada L.G. (2012). Counteracting Roles of AMP Deaminase and AMP Kinase in the Development of Fatty Liver. PLoS ONE.

[81] Yao Z., Vance D.E. (1990). Reduction in VLDL, but not HDL, in plasma of rats deficient in choline. Biochem. Cell Biol..

[82] Blumberg H., Mccollum E.V., Albanese A.A., Buschke W. (1941). The prevention by choline of liver cirrhosis in rats on high fat, low protein diets. Science.

[83] Sanders L.M., Zeisel S.H. (2007). Choline: Dietary Requirements and Role in Brain Development. Nutr. Today.

[84] Shaw G.M., Finnell R.H., Blom H.J., Carmichael S.L., Vollset S.E., Yang W., Ueland P.M. (2009). Choline and Risk of Neural Tube Defects in a Folate-fortified Population. Epidemiology.

[85] Rath S., Heidrich B., Pieper D.H., Vital M. (2017). Uncovering the trimethylamine-producing bacteria of the human gut microbiota. Microbiome.

[86] Zeisel S.H., Dacosta K.A., Youssef M., Hensey S. (1989). Conversion of Dietary Choline to Trimethylamine and Dimethylamine in Rats: Dose-Response Relationship. J. Nutr..

[87] Wang Z., Klipfell E., Wu Y., Schauer P., Smith J.D., Allayee H., Tang W.H.W., DiDonato J.A., Lusis A.J., Hazen S.L. (2011). Gut flora metabolism of phosphatidylcholine promotes cardiovascular disease. Nature.

[88] Chen Y.-M., Liu Y., Zhou R.-F., Chen X.-L., Wang C., Tan X.-Y., Wang L.-J., Zheng R.-D., Zhang H.-W., Ling W.-H. (2016). Associations of gut-flora-dependent metabolite trimethylamine-N-oxide, betaine and choline with non-alcoholic fatty liver disease in adults. Sci. Rep..

[89] Dumas M.-E., Barton R.H., Mitchell S.C., Holmes E., McCarthy M.I., Scott J., Gauguier D., Nicholson J.K., Toye A., Cloarec O. (2006). Metabolic profiling reveals a contribution of gut microbiota to fatty liver phenotype in insulin-resistant mice. Proc. Natl. Acad. Sci. USA.

[90] Gao X., Liu X., Xu J., Xue C., Xue Y., Wang Y. (2014). Dietary trimethylamine N-oxide exacerbates impaired glucose tolerance in mice fed a high fat diet. J. Biosci. Bioeng..

[91] Koeth R.A., Wang Z., Levison B.S., Buffa J.A., Org E., Sheehy B.T., Britt E.B., Fu X., Wu Y., Li L. (2013). Intestinal microbiota metabolism of l-carnitine, a nutrient in red meat, promotes atherosclerosis. Nat. Med..

[92] Baxter N.T., Schmidt A.W., Venkataraman A., Kim K.S., Waldron C., Schmidt T.M. (2019). Dynamics of Human Gut Microbiota and Short-Chain Fatty Acids in Response to Dietary Interventions with Three Fermentable Fibers. mBio.

[93] Perry R.J., Peng L., Barry N.A., Cline G.W., Zhang D., Cardone R.L., Petersen K.F., Kibbey R.G., Goodman N.A.B.A.L., Shulman R.J.P.L.P.G.W.C.R.L.C.K.F.P.R.G.K.G.I. (2016). Acetate mediates a microbiome–brain–β-cell axis to promote metabolic syndrome. Nat. Cell Biol..

[94] Vinolo M.A.R., Rodrigues H.G., Fock R.A., Malheiros G., Dos Santos M.F., Curi R., Festuccia W.T.L., Crisma A.R., Alves V.S., Martins A.R. (2012). Tributyrin attenuates obesity-associated inflammation and insulin resistance in high-fat-fed mice. Am. J. Physiol. Endocrinol. Metab..

[95] Weitkunat K., Stuhlmann C., Postel A., Rumberger S., Fankhänel M., Woting A., Petzke K.J., Gohlke S., Schulz T.J., Blaut M. (2017). Short-chain fatty acids and inulin, but not guar gum, prevent diet-induced obesity and insulin resistance through differential mechanisms in mice. Sci. Rep..

[96] Park J., Kim M., Kang S.G., Jannasch A.H., Cooper B., Patterson J., Kim C.H. (2015). Short-chain fatty acids induce both effector and regulatory T cells by suppression of histone deacetylases and regulation of the mTOR–S6K pathway. Mucosal Immunol..

[97] Kim M.H., Kang S.G., Park J.H., Yanagisawa M., Kim C.H. (2013). Short-Chain Fatty Acids Activate GPR41 and GPR43 on Intestinal Epithelial Cells to Promote Inflammatory Responses in Mice. Gastroenterology.

[98] Balmer M.L., Ma E.H., Bantug G.R., Grählert J., Pfister S., Glatter T., Jauch A., Dimeloe S., Slack E., Dehio P. (2016). Memory CD8+ T Cells Require Increased Concentrations of Acetate Induced by Stress for Optimal Function. Immunity.

[99] Müller M., Hernández M.A.G., Goossens G.H., Reijnders D., Holst J.J., Jocken J.W.E., Van Eijk H., Canfora E.E., Blaak E.E. (2019). Circulating but not faecal short-chain fatty acids are related to insulin sensitivity, lipolysis and GLP-1 concentrations in humans. Sci. Rep..

[100] Watanabe-Suzuki K., Seno H., Ishii A., Kumazawa T., Suzuki O. (1999). Ultra-sensitive method for determination of ethanol in whole blood by headspace capillary gas chromatography with cryogenic oven trapping. J. Chromatogr. B Biomed. Sci. Appl..

[101] Yuan J., Chen C., Cui J., Lu J., Yan C., Wei X., Zhao X., Li N., Li S., Xue G. (2019). Fatty Liver Disease Caused by High-Alcohol-Producing Klebsiella pneumoniae. Cell Metab..

[102] Jeon S., Carr R. (2020). Alcohol effects on hepatic lipid metabolism. J. Lipid Res..

[103] Raucy J.L., Lasker J., Ozaki K., Zoleta V. (2004). Regulation of CYP2E1 by Ethanol and Palmitic Acid and CYP4A11 by Clofibrate in Primary Cultures of Human Hepatocytes. Toxicol. Sci..

[104] Chen P., Miyamoto Y., Mazagova M., Lee K.-C., Eckmann L., Schnabl B. (2015). Microbiota Protects Mice Against Acute Alcohol-Induced Liver Injury. Alcohol. Clin. Exp. Res..

[105] Boyer J.L. (2013). Bile Formation and Secretion. Compr. Phys..

[106] David L.A., Maurice C.F., Biddinger S.B., Dutton R.J., Turnbaugh P.J., Carmody R.N., Gootenberg D.B., Button J.E., Wolfe B.E., Ling A.V. (2014). Diet rapidly and reproducibly alters the human gut microbiome. Nature.

[107] Copple B.L., Li T. (2016). Pharmacology of bile acid receptors: Evolution of bile acids from simple detergents to complex signaling molecules. Pharmacol. Res..

[108] Sinal C.J., Tohkin M., Miyata M., Ward J.M., Lambert G., Gonzalez F.J. (2000). Targeted Disruption of the Nuclear Receptor FXR/BAR Impairs Bile Acid and Lipid Homeostasis. Cell.

[109] Keitel V., Donner M., Winandy S., Kubitz R., Häussinger D. (2008). Expression and function of the bile acid receptor TGR5 in Kupffer cells. Biochem. Biophys. Res. Commun..

[110] Neuschwander-Tetri B.A., Loomba R., Sanyal A.J., Lavine J.E., Van Natta M.L., Abdelmalek M.F., Chalasani N., Dasarathy S., Diehl A.M., Hameed B. (2015). Farnesoid X nuclear receptor ligand obeticholic acid for non-cirrhotic, non-alcoholic steatohepatitis (FLINT): A multicentre, randomised, placebo-controlled trial. Lancet.

[111] Moro-Sibilot L., Blanc P., Taillardet M., Bardel E., Couillault C., Boschetti G., Traverse-Glehen A., Defrance T., Kaiserlian D., Dubois B. (2016). Mouse and Human Liver Contain Immunoglobulin A–Secreting Cells Originating From Peyer’s Patches and Directed Against Intestinal Antigens. Gastroenterology.

[112] Brown W.R., Kloppel T.M. (1989). The role of the liver in Translocation of IgA into the Gastrointestinal Tract. Immunol. Investig..

[113] Rogier E.W., Frantz A.L., Bruno M.E.C., Wedlund L., Cohen D.A., Stromberg A.J., Kaetzel C.S. (2014). Secretory antibodies in breast milk promote long-term intestinal homeostasis by regulating the gut microbiota and host gene expression. Proc. Natl. Acad. Sci. USA.

[114] Juo Y.-Y., Livingston E.H. (2019). Testing for Nonalcoholic Fatty Liver Disease. JAMA.

[115] Rinella M.E. (2015). Nonalcoholic Fatty Liver Disease. JAMA.

[116] Lee G., You H.J., Bajaj J.S., Joo S.K., Yu J., Park S., Kang H., Park J.H., Kim J.H., Lee D.H. (2020). Distinct signatures of gut microbiome and metabolites associated with significant fibrosis in non-obese NAFLD. Nat. Commun..

[117] Caussy C., Hsu C., Schork N., Schnabl B., Brenner D.A., Sirlin C.B., Chen C.-H., Loomba R., Lo M.-T., Genetics of NAFLD in Twins Consortium (2018). Link between gut-microbiome derived metabolite and shared gene-effects with hepatic steatosis and fibrosis in NAFLD. Hepatology.

[118] Bajaj J.S., Salzman N.H., Lee H., Osman M., Siddiqui M.S., Fuchs M., Puri P., Sikaroodi M., Gillevet P.M., Acharya C. (2019). Fecal Microbial Transplant Capsules Are Safe in Hepatic Encephalopathy: A Phase 1, Randomized, Placebo-Controlled Trial. Hepatology.

[119] Philips C.A., Pande A., Shasthry S.M., Jamwal K.D., Khillan V., Chandel S.S., Kumar G., Sharma M.K., Maiwall R., Jindal A. (2017). Healthy Donor Fecal Microbiota Transplantation in Steroid-Ineligible Severe Alcoholic Hepatitis: A Pilot Study. Clin. Gastroenterol. Hepatol..

[120] Degnan F.H. (2012). Clinical studies involving probiotics. Gut Microbes.

[121] Gibson G.R., Roberfroid M.B. (1995). Dietary Modulation of the Human Colonic Microbiota: Introducing the Concept of Prebiotics. J. Nutr..

[122] Vamanu E. (2019). Complementary Functional Strategy for Modulation of Human Gut Microbiota. Curr. Pharm. Des..

[123] Gibson G.R., Hutkins R., Sanders M.E., Prescott S.L., Reimer R.A., Salminen S.J., Scott K., Stanton C., Swanson K.S., Cani P.D. (2017). Expert consensus document: The International Scientific Association for Probiotics and Prebiotics (ISAPP) consensus statement on the definition and scope of prebiotics. Nat. Rev. Gastroenterol. Hepatol..

[124] Sharpton S.R., Maraj B., Harding-Theobald E., Vittinghoff E., Terrault N.A. (2019). Gut microbiome–targeted therapies in nonalcoholic fatty liver disease: A systematic review, meta-analysis, and meta-regression. Am. J. Clin. Nutr..

[125] Duan Y., Llorente C., Lang S., Brandl K., Chu H., Jiang L., White R.C., Clarke T.H., Nguyen K., Torralba M. (2019). Bacteriophage targeting of gut bacterium attenuates alcoholic liver disease. Nature.

[126] Wang Z., Roberts A.B., Buffa J.A., Levison B.S., Zhu W., Org E., Gu X., Huang Y., Zamanian-Daryoush M., Culley M.K. (2015). Non-lethal Inhibition of Gut Microbial Trimethylamine Production for the Treatment of Atherosclerosis. Cell.

[127] Cipriani S., Mencarelli A., Palladino G., Fiorucci S. (2010). FXR activation reverses insulin resistance and lipid abnormalities and protects against liver steatosis in Zucker (fa/fa) obese rats. J. Lipid Res..

[128] Fickert P., Fuchsbichler A., Moustafa T., Wagner M., Zollner G., Halilbasic E., Stöger U., Arrese M., Pizarro M., Solís N. (2009). Farnesoid X Receptor Critically Determines the Fibrotic Response in Mice but Is Expressed to a Low Extent in Human Hepatic Stellate Cells and Periductal Myofibroblasts. Am. J. Pathol..

[129] Verbeke L., Farre R., Laleman W., Trebicka J., Komuta M., Roskams T., Klein S., Elst I.V., Windmolders P., Vanuytsel T. (2014). Obeticholic acid, a farnesoid X receptor agonist, improves portal hypertension by two distinct pathways in cirrhotic rats. Hepatology.

[130] Harrison S.A., Rinella M.E., Abdelmalek M.F., Trotter J.F., Paredes A.H., Arnold H.L., Kugelmas M., Bashir M.R., Jaros M.J., Ling L. (2018). NGM282 for treatment of non-alcoholic steatohepatitis: A multicentre, randomised, double-blind, placebo-controlled, phase 2 trial. Lancet.

